# IFN-Alpha-Mediated Differentiation of Dendritic Cells for Cancer Immunotherapy: Advances and Perspectives

**DOI:** 10.3390/vaccines8040617

**Published:** 2020-10-19

**Authors:** Caterina Lapenta, Lucia Gabriele, Stefano Maria Santini

**Affiliations:** Department of Oncology and Molecular Medicine, Istituto Superiore di Sanità, Viale Regina Elena 299, 00161 Rome, Italy; caterina.lapenta@iss.it

**Keywords:** interferon, dendritic cells, cancer vaccines

## Abstract

The past decade has seen tremendous developments in novel cancer therapies through targeting immune-checkpoint molecules. However, since increasing the presentation of tumor antigens remains one of the major issues for eliciting a strong antitumor immune response, dendritic cells (DC) still hold a great potential for the development of cancer immunotherapy. A considerable body of evidence clearly demonstrates the importance of the interactions of type I IFN with the immune system for the generation of a durable antitumor response through its effects on DC. Actually, highly active DC can be rapidly generated from blood monocytes in vitro in the presence of IFN-α (IFN-DC), suitable for therapeutic vaccination of cancer patients. Here we review how type I IFN can promote the ex vivo differentiation of human DC and orientate DC functions towards the priming and expansion of protective antitumor immune responses. New epigenetic elements of control on activation of the type I IFN signal will be highlighted. We also review a few clinical trials exploiting IFN-DC in cancer vaccination and discuss how IFN-DC could be exploited for the design of effective strategies of cancer immunotherapy as a monotherapy or in combination with immune-checkpoint inhibitors or immunomodulatory drugs.

## 1. Introduction

Cancer immunotherapy is typically aimed at stimulating or enhancing antitumor immune response in oncological patients. Among different immunotherapeutic approaches, therapeutic cancer vaccines are designed to instruct the immune system to identify and eradicate tumor cells, while preserving normal cells and tissues from immune attack, presumably preventing undesirable side effects. Cancer vaccines have the potential to control tumors as monotherapy or in combination with other forms of immunotherapy, as well as with nonimmune-based therapies, such as radiotherapy or chemotherapy. In particular, in patients with minimal residual disease after tumor debulking, this therapeutic option may result in prolonged survival and improved life quality. As a consequence of the recent success of immune checkpoint inhibitors (ICI) in the treatment of cancer patients [1], dendritic cells (DC), specialized in sensitizing lymphocytes to tumor antigens, have gained renewed interest as critical cell adjuvants in immunotherapeutic approaches. In particular, DC-based vaccines and T-cell checkpoint blockade can act as synergistic partners, as checkpoint inhibitors simply function as boosters of immune responses and their efficacy is proportional to the pre-existing amount of tumor-specific T cells at the tumor site.

## 2. The Link between Type I IFN and DC in Cancer Rejection

DC are professional antigen presenting cells (APC), acting at the interface between the environment and the immune system and bridging the gap between innate and adaptive immunity [2]. By virtue of their unique ability to take up and process antigens in the peripheral blood and tissues, DC play a crucial role in the initiation of primary immune responses. Upon maturation/activation, DC undergo phenotypic changes, increase MHC and costimulatory molecule expression, and upregulate cytokine production. Mature DC promptly migrate to draining lymph nodes, to prime naïve T cells and initiate adaptive immune response [2]. Since their discovery, it has been shown that DC lineage is complex and includes a variety of different subsets: conventional DC (cDC), plasmacytoid DC (pDC), Langerhans cells and monocyte-derived DC (moDC). DC have attracted considerable attention as potential cell-drugs in the preparation of therapeutic cancer vaccines. Cancer vaccination has been performed using reinfusion of defined populations of DC obtained ex vivo from peripheral blood, including the use of BDCA1+ cDC and pDC [3,4,5]. However, the scarceness of these DC subsets in the peripheral blood has so far imposed major limitations to their use in the clinical setting. Therefore, most DC-based vaccines have exploited moDC differentiated ex vivo from monocytes cultured in the presence of IL-4 and GM-CSF or other cytokines, because of the relative ease of recovering large numbers of these cells from the peripheral blood. However, the choice of an optimal protocol of DC generation in vitro for the preparation of clinically effective therapeutic cancer vaccines still represents a major challenge. While the optimal culture conditions for generating the most effective moDC is still controversial, some groups, including ours, have shown that partially mature and highly active moDC from blood monocytes can be rapidly generated in the presence of IFN-α and GM-CSF (IFN-DC) [6,7].

Although type I IFN (IFN-α and IFN-β; hereafter IFN-I) was originally characterized for its antiviral activity [8], it is also known to mediate antiproliferative and antineoplastic effects and proved the most useful and wide-ranging biologic agent against several tumors [9]. IFN-α has been used for the treatment of selected tumors, including melanoma and renal cancer, showing its best efficacy in hematological malignancies, such as hairy cell leukemia, chronic myeloid leukemia, and follicular lymphoma [10]. Direct evidence of IFN-α activity in both B and T-cell low-grade lymphomas is the regression of cutaneous and conjunctival neoplastic lesions following repeated in situ injections of this pleiotropic drug [11,12]. In solid tumors the results have been more disappointing. However, evidence exists showing that IFN-α can be beneficial against early stage cancers, but much less effective against established or metastatic tumors [13]. Due to adverse effects of systemic high dose IFN-α administration in cancer patients and the development of more effective drugs and protocols, the initial interest in IFN-based therapies rapidly faded down. Conversely, a growing interest has emerged on the immunomodulatory role of type I IFN, since a considerable body of evidence clearly demonstrates that IFN-α can bridge innate and adaptive immunity through its effects on DC differentiation/activation, skewing DC functions towards the priming and expansion of protective antitumor immune responses [14,15]. Studies to evaluate the direct effect of IFN-α on experimental and conventional vaccines in mice and humans have also been performed [16]. Nevertheless, a few pilot studies have also attempted to evaluate the possible immune modulating activity of these cytokines in vaccination strategies. In some of them, IFN-α induced improved immunological responses [17,18,19] or enhanced peptide immunogenicity [20,21,22]. On the whole, these findings are strongly consistent with studies performed over the last 20 years, showing the importance of IFN-α driven generation of highly active DC and the induction of adaptive immunity. Le Bon et al. demonstrated that DC were the cell type mediating the adjuvant effect of IFN-I in vivo, inducing long-term antibody production and immunological memory against a poorly immunogenic antigen [23]. In addition, DC activation by IFN-I can promote spontaneous immune responses to tumor cells, including the cross-priming of tumor-specific CD8 T cells, [24,25,26,27]. Therefore, the immune response for cancer rejection appears to exploit inflammatory mechanisms reminiscent of those activated in early antiviral defense mediated by IFN-I release from pDC and macrophages [28]. In this view, IFN-α may be involved in a proinflammatory condition promoting the in vivo conversion of monocytes into DC, initiating antiviral and antitumor specific immune responses. In fact, significant amounts of IFN-I can be locally released at the site of infection or inflammation. This may enable the differentiation of circulating monocytes into activated DC mediating the activation of natural killer cells, the generation of a Th1-polarized T-helper response, and the induction of a cytolytic response against both viruses and cancer cells. Worth mentioning, infiltrating IFN-DC have been demonstrated in regressing molluscum contagiosum skin lesions, characterized by the accumulation of pDC and the local production of IFN-I [29]. Reasonably, the culture conditions developed for the generation of IFN-DC in vitro may reproduce the natural cytokine milieu enabling the rapid differentiation of DC from monocyte in vivo and could be considered a physiological pathway of monocyte conversion into DC.

## 3. IFN-α-Conditioned Dendritic Cells (IFN-DC)

IFN-α and IFN-β differently modulate DC activation/maturation, depending on the experimental model and culture conditions. Indeed, IFN-α has been shown to markedly enhance DC maturation [30,31,32]. Moreover, IFN-α can synergize with polyinosinic:polycytidylic acid (p-I:C) and the “classical” type-1-polarizing cytokine cocktail, allowing for serum-free generation of fully mature type-1-polarized DC (DC1) [33,34], providing DC with different chemoattractive properties [35]. However, in 1998 it became apparent that IFN-α in itself was capable of driving the differentiation of blood monocytes into DC [36]. Soon after, our group reported that a three day culture in the presence of IFN-α and GM-CSF can convert blood monocytes into fully functional and partially mature DC (IFN-DC), without the addition of maturation factors or further culture steps [6,7]. Since then, numerous studies have confirmed that IFN-I can efficiently induce the differentiation of blood monocytes into DC favoring Th1 biased response, huge production of IFN-γ, and the efficient expansion of CD8 effector T cells [37,38,39,40,41]. As a result of IFN-α transcriptional signature, IFN-DC exhibit distinct molecular and functional features, showing a more advanced maturation phenotype, as compared to conventional moDC obtained with IL-4 and GM-CSF, with the expression of higher levels of costimulatory molecules as well as variable amounts of the maturation marker CD83 [6,7]. They also display mixed features of natural killer (NK cells) and pDC with significant levels of CD123 [7,29,38].

IFN-DC can efficiently initiate an adaptive immune response by virtue of the high expression of some important molecules involved in antigen processing, migration, and localization in the lymph nodes [28,29]. IFN-DC are endowed with improved migratory response to chemokines and express very high levels of CCR5. They exhibit an enhanced response to its ligands CCL5, CCL3, CCL4 as well. A considerable fraction of IFN-DC also expresses integrin α4 and CCR7 [42].

Moreover, IFN-DC demonstrate an improved migratory response to CCL19 and express significant levels of CCL19 themselves, together with CCL18 and CXCL10 [42]. Of note, high levels of monocyte chemoattractant proteins (MCPs), CXCL2 and CXCL-3 confer IFN-DC the capability to efficiently mediate the recruitment of other innate effector cells as well as a Th1-skewed cytokine production [38,43]. Despite their advanced maturation state, IFN-DC retain an efficient phagocytic activity [7], promptly acquiring a fully mature phenotype upon interaction with peripheral blood lymphocytes (PBL) [44]. IFN-DC can take up apoptotic cells through the scavenger receptor lectin-like oxidized-LDL receptor-1 (LOX-1) and cross-present their antigens to CD8+ T cells. [45]. IFN-DC are also directly licensed for CD4-independent CD8+ T cell priming, targeting antigen onto class I molecules, cross-presenting very efficiently low amounts of soluble proteins to CD8+ T cells [46]. Both immature and mature IFN-DC express high amounts of immunoproteasome subunits (LMP2, LMP7, and MECL1) along with elevated levels of TAP1, TAP2, calnexin, calreticulin, tapasin, and HLA class I molecules [47,48]. This functional attitude of IFN-DC results in very efficient triggering of specific CD8 T lymphocytes, specific for a subdominant MHC-I-restricted viral epitope and MART-127–35 epitope [48]. Noteworthy, the improved capacity of IFN-DC to protect internalized proteins from early degradation and to efficiently route antigens toward the MHC-I processing pathway, allows a long-lasting cross-priming capacity [49]. This suggests the potential ability of IFN-DC to retain antigens for an extended period in lymph nodes after their uptake, allowing the encounter and recruitment of rare specific CD8+ T-cell precursors, with important implications for the development of DC-based therapeutic vaccines. Importantly, IFN-DC also drive priming of naïve CD4 T cells, resulting in a massive expansion of CXCR3+ IFN-γ-producing CD4 Th1 cells [50]. IFN-DC express high levels of Fas-L and TRAIL, performing a direct tumoricidal activity [6,38,39,44]. Likewise, an important role of transmembrane TNF-α as mediator of IFN-DC killer activity, which becomes defective in high grade glioma patients, has been recently described [51]. The direct cytotoxic activity of IFN-DC against tumor cells represents an important functional feature, since it may facilitate tumor antigen uptake, resulting in earlier and improved induction of antitumor immune response.

## 4. Epigenetic Control of Gene Regulation in IFN-DC

In recent times, it has become evident that the epigenetic reprogramming drives the acquisition of specific functions of many immune populations, including DC, by simultaneous activation and repression of genes belonging to crucial cellular pathways. These events become particularly relevant for shaping trained immunity of DC, characterized by the persistence of transcriptional memory and the capability of more efficient functional responses [52]. IFN-I has the potential to epigenetically regulate the transcriptional activation of DC, inducing memory-like responses [53]. The regulation of microRNA expression as well as the incorporation of long-lasting chromatin marks, such as the histone variant H3.3 and the histone modification H3K36me3, represent major mechanisms operated by IFN-I to modulate the expression of interferon-stimulated genes (ISG) and inflammatory non-ISG [54]. From a molecular point of view, IFN-DC exhibit strong similarity with pDC, that are mainly blood DC specialized in the production of IFN-α in response to viruses and other danger signals [55]. IFN-DC, similarly to in vitro IFN-α-treated pDC, are outlined by a specific miRNA signature, with high expression of miR-23a and miR-125b, negatively associated with up-modulation of Blimp-1, a master regulator of effector and memory differentiation in B cells as well as in CD4+ and CD8+ T cells [56]. Of interest, IFN-DC and pDC share similar phenotypic and functional hallmarks further supporting the existence of a potential relationship between these DC populations. PDC were used successfully in clinical trials [56].

## 5. IFN-α-Conditioned DC in Cancer Immunotherapy

As immune responses with strong Th1 bias are considered adequate for the induction of optimal antitumor immunity, IFN-DC represent highly promising candidates for the development of DC-based immunotherapy trials in cancer (major preclinical and clinical studies testing IFN-DC in cancer immunotherapy are summarized in Table 1). In this regard, spontaneous regressing Molluscum Contagiosum Virus (MCV)-induced tumor-like lesions were shown to be heavily infiltrated with DC closely resembling IFN-DC [29]. However, only a few studies have been performed to establish their potential in the induction of an immune response to solid tumors (Table 1). In renal cell carcinoma (RCC) patients, IFN-DC were described to promote significantly higher numbers of autologous cytotoxic antitumor responses in vitro, as compared to classic DC, as well as to reduce regulatory-type T cells (Tregs) among CD4^+^ T-cell responder populations [57]. In 2008, Papewalis and colleagues reported on a small number of cancer patients with metastasized medullary thyroid carcinoma immunized with Ag-pulsed IFN-DC [39], showing an increase of Ag-specific IFN-γ-secreting T cells, a Th1-cytokine pattern, and delayed-type hypersensitivity reaction following injection with calcitonin-pulsed DC, with dense infiltration with CD8+ cells as assessed by immunohistochemical analyses. An interesting approach of endogenous vaccination with unloaded IFN-DC was tested in six advanced melanoma patients in a phase I clinical study of chemo-immunotherapy. The strategy exploited IFN-DC capacity to efficiently take up apoptotic tumor cells and soluble antigens in vivo. Treatment regimen consisted in six intratumoral injections of 10 million unloaded IFN-DC one day after administration of dacarbazine every 3 weeks. Both preconditioning and IFN-DC injection were well tolerated and no severe side effects were reported. Three patients showed temporary disease stabilization and two of them developed T cell response against tyrosinase, NY-ESO-1 and gp100 proteins. Long-lasting disease stabilization was seen in a single patient showing tyrosinase-specific T cell response as well persistent tumor infiltration by memory T cells [58].

Evidence of the effectiveness of monocyte conditioning with IFN-α and GM-CSF in immunotherapy has been provided by a number of studies in hematological malignancies. While IFN-α has been reported to induce a graft-versus-leukemia effect (GVL) when administered with donor leukocyte infusion (DLI) in patients who relapsed after allogeneic transplantation [59], the combination of IFN-α with GM-CSF can effectively drive the differentiation of monocytes from leukemic patients into DC-like-cells promoting anti-leukemic cytotoxicity [60,61,62]. In particular, the treatment of CML monocytes with IFN-α and GM-CSF allowed the rapid generation of activated IFN-DC expressing IL-15, which induced IFN-γ production by cocultured autologous T lymphocytes and stimulated the expansion of CD8+ T cells, which were reactive against autologous leukemic cells [60]. More recently, studies from our laboratory and others have shown IFN-DC as a promising tool for the development of DC-based immunotherapy for non-Hodgkin lymphomas (NHL). IFN-DC loaded with an immunogenic tumor cell lysate can elicit lymphoma-specific CTL in an experimental model of mantle cell lymphoma (MCL) and diffuse large B-cell lymphoma (DLBCL) [63]. Lysate loading onto IFN-DC resulted in enhanced functional maturation and activation. Furthermore, treatment of hu-PBL-NOD/SCID mice with the IFN-DC vaccine was able to inhibit lymphoma growth. The high titers of IFN-γ in the sera of vaccinated mice was consistent with the ability IFN-DC to induce a systemic Th1-skewed immune response while an in vivo antitumor immune response was shown to be mediated by both Th1 and Th17 cells [63]. We also evaluated an in vitro vaccination procedure based on IFN-DC loaded with lymphoma cells undergoing immunogenic apoptosis [64]. Of note, we showed that apoptotic tumor cell-loaded IFN-DC from follicular lymphoma (FL) patients, cultured with autologous lymphocytes, led to Th1 polarization and an improved cellular response [64]. The hallmark of the response elicited was a remarkable increase in CD8 T cells and an early massive NK cell activation with increased expression of cytotoxicity receptors and extensive IFN-γ production. Consistent with the detection of enhanced cytotoxic effector function toward autologous FL cells. Importantly, a critical role for MICA/B and membrane-bound IL-15 in IFN-DC-mediated NK cell activation and early IFN-γ production as well as direct recognition and killing of primary autologous lymphoma cells by activated NK cells was demonstrated [64]. In accordance with other studies [39,57], we also showed IFN-DC as poor inducers of regulatory T cells (Tregs) expansion and suppressive functions. Taken together, these results indicated that apoptotic lymphoma cells represent an optimal antigenic formulation for IFN-DC loading. In contrast to anti-idiotype vaccination, this strategy offers the additional advantage of presenting multiple tumor Ag contained within FL cells, thus enabling a wider and more efficient antitumor immune response.

The efficacy of in vivo therapeutic vaccination with IFN-DC was subsequently demonstrated in the xenochimeric mouse model of NOD/SCID mice reconstituted with human PBL [65]. Significant inhibition of tumor cell growth and spread to lymph nodes in hu-PBL-NOD/SCID mice bearing established human follicular lymphoma was observed after repeated cycles of therapeutic vaccination with apoptotic tumor cell-loaded IFN-DC. Notably, the combination of IFN-DC-based vaccination plus lenalidomide exhibited an additive therapeutic effect, resulting far more effective than either single treatment, leading to a massive regression of established tumors and delayed tumor regrowth upon treatment discontinuation.

The above studies supported the start of a phase I clinical study of therapeutic vaccination of refractory and relapsed FL patients [44]. The trial was based on the assumption of endogenous vaccination acting through repeated waves of rituximab-induced lymphoma cell killing, followed by the Fc-receptor-mediated uptake of tumor-associated antigens by IFN-DC exploiting drug-induction of tumor cell death with release of tumor-associated antigens. Low-dose rituximab followed 24 h later by IFN-DC were administered by direct intranodal injection in one affected superficial lymphoma node, applied in a every 2 week regimen for the first four treatment cycles and monthly for the remaining four cycles. Although the limited number of patients evaluated in the trial did not allow drawing any definitive conclusion, this study provided the first evidence of the safety and clinical efficacy of IFN-DC with an overall response rate (ORR) of 50%. Impressive regression of untreated lymphoma lesions distal to the injection site was observed, suggesting the occurrence of a systemic response to endogenous vaccination. Four patients who experienced partial or complete clinical responses also showed lymphoma-specific responses directed toward both class-I and class-II restricted clonal idiotype epitopes peaking at 6–9 months from the start of treatment [44].

## 6. Perspectives of Combinatorial Immunotherapy Regimens with IFN-DC Vaccines

Considering the importance of the immune response in the evolution of cancer, the development of immunotherapeutic strategies has become a major field of research in recent decades, especially those aimed at targeting inhibitory immune checkpoint molecules. Currently, the most dominant therapeutic strategy with immune checkpoint inhibitors (ICI) in clinical trials is that targeting the PD-1-PD-L1 axis. On the whole, 11 ICI have been approved in treating 16 types of malignant diseases [66]. Yet, we believe that there still room for cancer vaccines at the era of ICI, especially in minimal residual disease, to clear residual cancer and prevent tumor relapse.

**Figure 1:**
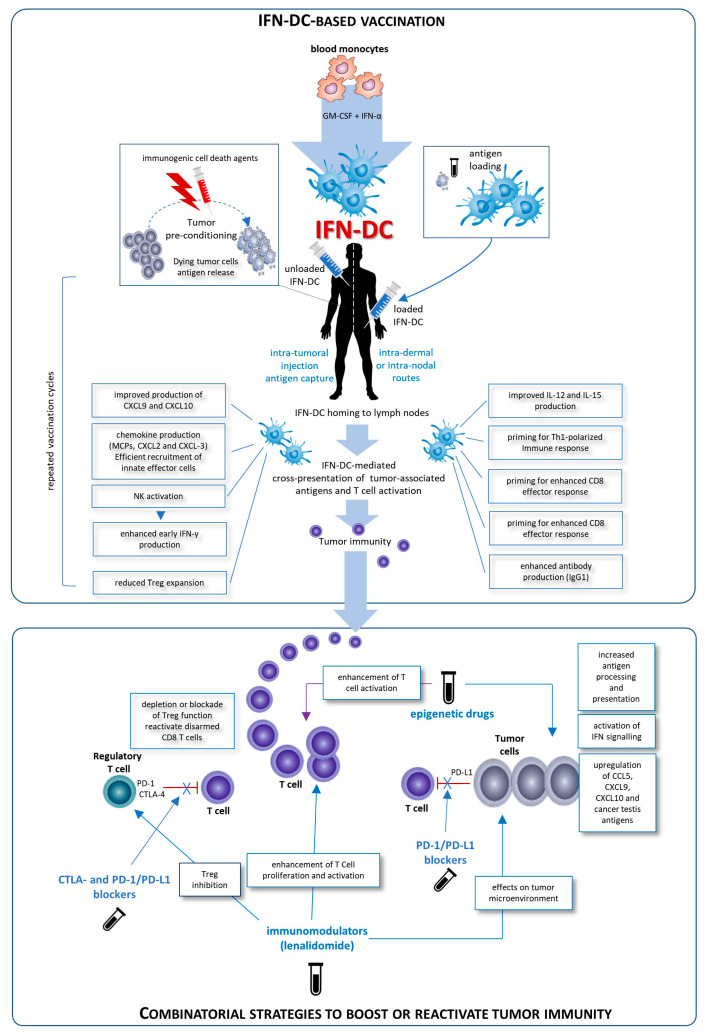
Possible clinical exploitation of IFN-DC in cancer vaccination. Patients undergo leukapheresis to collect PBMC and purify blood monocytes necessary for IFN-DC generation. Large numbers of partially mature IFN-DC can be easily obtained at one-time-point from purified peripheral blood monocytes cultured in the presence IFN-α and GM-CSF, loaded or not with tumor antigens, and cryopreserved in ready-for-use aliquots for the programmed cycles of treatment. On the left is depicted the prototypical intratumoral vaccination strategy based on the concept of tumor preconditioning with immunogenic cell death agents followed by unloaded IFN-DC. The intratumoral injections are guided by ultrasound and performed by a radiologist to ensure correct administration. In the right of the figure, the therapeutic vaccination strategy is shown. IFN are loaded in vitro with selected formulation of tumor antigens and administered intradermally, in close vicinity to axillary and inguinal lymph nodes or directly administered into a healthy lymph node. In both strategies the treatment cycles are repeated at two-week intervals. IFN-DC are characterized by the capacity to release a unique array of cytokines and chemokines known to favor Th1 type response and to powerfully stimulate cellular CD8^+^ T cell immune responses as well as to promote IgG1 isotype antibodies response [6,7,42,50,64].

Despite IFN-DC have been proved effective in generating T cell responses against solid tumors and lymphomas, the full potential of this immunotherapeutic strategy will be exploited in combination therapies, in order to generate tumor-specific immune responses associated with long-term survival. Indeed, cancer vaccines may benefit from the synergistic combination with other types of treatment aimed at relieving constraints imposed by tumor-induced immunosuppression (Figure 1). Paradoxically, the upregulation of PD-L1, indoleamine-2,3-dioxygenase (IDO), and Tregs in tumor microenvironment has been shown to be driven by IFN-γ-producing CD8+ T cells themselves [67,68] and these three factors can all contribute to disable T cell responses and impair vaccine efficacy [69]. On the other hand, limitations of checkpoint immunotherapy actually exist. Despite promising results with ICI, PD-1 inhibitors have an objective response rate that varies from 50% to 80% in melanoma, Merkel cell carcinoma and squamous-cell carcinoma to an average of 15–30% in most other cancers, while virtually no improvements have been seen in tumors like pancreatic cancer [70]. Combination of PD-1 blockers with other ICI can improve the response rate, but with unacceptable higher toxicity related to immune adverse events. Since ICI require pre-existing antitumor T cells at the tumor site and their clinical efficacy depends on the extent of T cell infiltration [71], their combination with cancer vaccines is an obvious strategy to pursue, in order to sensitize the host’s immune system to the tumor in advance (Figure 1), without increasing autoimmunity.

Of particular importance is the role of cancer vaccination in tumors with no anticancer immunity, owing to low mutational burden, defects in cancer antigen release or presentation as well as to tumor-induced immunosuppression. T-cell-inflamed tumors are characterized by signatures of immune activation, type I IFN transcriptional profile, as well as extensive T cell infiltration, which have been associated with clinical response to checkpoint blockade [27]. Nevertheless, it has been shown that about 70% of cancers are not significantly infiltrated by CD8+ T cells [72]. A defect that active DC-based vaccination typically aims to correct, potentially converting a “cold” tumor refractory to checkpoint inhibitor blockade into a sensitive T-cell-inflamed tumor [27]. Of note, while a defective differentiation and functional alteration of the endogenous DC has been observed in cancer patients [73], the injection of autologous antigen-pulsed or unloaded DC, generated ex vivo, may circumvent tumor-induced dysfunction and restore immune responses. Moreover, accumulating evidence suggests that DC recruitment and crosstalk with T cells is critically required for anti-PD-1-mediated antitumor response [74,75]. Importantly, the chance to integrate cancer vaccines in future combinatorial immunotherapy regimens extends beyond checkpoint inhibitors to include immune costimulatory agonists (i.e., OX40 and 4-1BB), immunomodulatory agents, as well as selected inhibitors of oncogenic kinases (i.e., BRAF and MEK) [76]. Hence, in the next years, we need to assess the clinical effectiveness of different combinatorial strategies to increase efficacy of cancer immunotherapy.

Our recent findings suggest that IFN-DC are a good candidate for a vaccinal clinical use in cancer patients. Basically, two major modalities for the development of novel IFN-DC-based therapies can be envisaged: the standard administration of IFN-DC loaded with autologous tumor cells and intratumoral vaccination based on the concept of tumor preconditioning with immunogenic cell death agents followed by unloaded IFN-DC (Figure 1). Both approaches would finally culminate in the cross-presentation of tumor-associated antigens to CD8 T-cells and their activation. Importantly, IFN-DC loaded ex vivo or in vivo with whole tumor cells offer the advantage of eliciting immunity against the entire collection of antigens expressed by the tumor, enabling a wider and more efficient antitumor immune response. In this regard, IFN-DC-vaccine based on whole tumor-cells induced to undergo immunogenic cell death can represent an optimal antigenic formulation for IFN-DC loading [63,64,65]. Interestingly, it has been recently shown that autophagosomes can be an excellent antigenic formulation to load IFN-DC, capable of inducing improved T cell response and IFN-y secretion as compared to cDC [77].

Since increasing the presentation of tumor antigens remains one of the major issues for eliciting a competent and strong antitumor immune response, great attention is paid to reprogramming the environment of tumor-associated immunity by pharmacologic modulation of epigenetic modifications (Figure 1). On this line, encouraging results come from recent preclinical studies and clinical trials focused on the optimization of enhanced antitumor response rates by combining epigenetic agents and ICI [78]. Likewise, a pivotal clinical trial is testing the combination of the epigenetic drug guadecitabine with a DC-based vaccine against the cancer testis antigen NY-ESO-1, in patients with recurrent ovarian and primary peritoneal cancer [79].

The value of combining IFN-DC with other agents has been extensively demonstrated by our recent therapeutic approach with IFN-DC-based lymphoma vaccine and the immunomodulatory drug lenalidomide in the treatment of xenochimeric mice bearing established human lymphoma, resulting in a massive regression of tumor masses and long-lasting inhibition of tumor regrowth after treatment discontinuation, over the single treatments [65]. Lenalidomide has been reported to synergize with rituximab by enhancing NK-mediated ADCC and lymphoma cell killing through complementary mechanisms, [80]. Thus, a useful strategy for increasing antigen availability and uptake by IFN-DC would be to combine intratumoral rituximab and IFN-DC plus systemic lenalidomide, in order to improve the cross-presentation of lymphoma antigens to CD8+ cells.

## 7. Conclusions

Data provided by preclinical and early clinical pilot studies indicate that IFN-DC vaccination can induce immunological as well as clinical responses in cancer patients. However, further clinical studies are currently needed to give IFN-DC reliability as a new option in cancer vaccination. A successful cellular vaccine should be easy to manufacture in a reproducible manner from most appropriate DC precursors. Fully functional IFN-DC are differentiated in just 3 days of culture without further culture steps in the absence of maturation factors and cryopreserved in aliquots for clinical application. Actually, there are no major limitations for the clinical exploitation of IFN-DC, as large numbers of semi-mature IFN-DC can be easily obtained at one time point from peripheral blood monocytes purified from leukapheresis product.

**Table 1:** Major preclinical and clinical studies testing IFN-DC in cancer immunotherapy.

Tumor Setting	DC Features	Type of Study	Major Findings	Refer/Year
Chronic myeloid leukemia (CML)	Generation of activated IFN-DC from CML monocytes	Preclinical	Expansion of CML-specific CD8+ T cells	[60] Gabriele 2004
Renal cell carcinoma (RCC)	Peptide-pulsed IFN-DC (HLA-A2 restricted peptides)	Preclinical	Induction of specific cytotoxic T cells; low levels of Tregs	[57] Gigante 2008
Metastasized medullary thyroid carcinoma	Calcitonin-pulsed IFN-DC	Clinical	Induction of IFN-γ-secreting T cells, a Th1-cytokine pattern and DTH reaction	[39] Papewalis 2008
Melanoma and Lymphoblastoid Cell Lines (LCL)	IFN-DC loaded with peptides, tumor cell lysate or apoptotic cells	Preclinical	Stimulation of CTL effector upon cross-presentation of specific epitopes.	[48] Lattanzi 2011
Melanoma	Unloaded IFN-DC-preconditioning with dacarbazine	Clinical	Systemic antitumor immune response; temporary disease stabilization	[58] Rozera 2015
Follicular lymphoma (FL)	IFN-DC loaded with apoptotic FL cells	Preclinical	Th1-skewed immune response, enhanced cytotoxic response	[64] Lapenta 2016
Mantle cell lymphoma (MCL), diffuse large B-cell lymphoma (DLBCL)	IFN-DC loaded with tumor cell lysate.	Preclinical	Th1-skewed immune response; in vivo lymphoma growth inhibition in hu-PBL-NOD/SCID mice	[63] Montico 2017
High-grade glioma	Unloaded IFN-DC	Preclinical	Correction of defective IFN-DC tumoricidal activity by treatment with IL-2 or Double-Stranded DNA	[51] Tyrinova 2017
Follicular lymphoma (FL)	IFN-DC loaded with apoptotic FL cells	Preclinical	Inhibition of tumor cell growth and spread in hu-PBL-NOD/SCID mice	[65] Lapenta 2019
Stage III-IV follicular lymphoma (FL)	Preconditioning with low-dose intratumoral Rituximab-unloaded IFN-DC	Clinical	Specific CD8+ and CD4 T-cell; regression of untreated lymphoma lesions	[44] Cox 2019
Acute Myeloid Leukemia (AML)	Unloaded IFN-DC from AML-blasts	Preclinical	Improved T cell anti-leukemic cytotoxicity	[61] Hirn Lopez 2019

Conceivably, IFN-DC-based monotherapy can evolve in chemotherapy-free combinatorial therapy regimens with immune-checkpoint inhibiting antibodies as well as immunomodulating or epigenetic drugs. The blockade of inhibitory pathways or activation promotes CD8 T cell priming after vaccination. Inhibition of Treg alleviates the suppressor activity of these cells on effector CD8+T cells. Blockade of the interaction of PD-1/PD-L1 reactivates disarmed CD8 cells and antitumor effector functions. An attractive immunomodulatory drug to be combined with IFN-DC-based therapies is lenalidomide, as it acts through the boosting of antitumor immunity and the modification of tumor microenvironment. Additionally, epigenetic therapies for cancer including DNA methyltransferase inhibitors (DNMTi), histone deacetylase inhibitors (HDACi), and histone methyltransferase inhibitors (HMTi) can stimulate antitumor immunity in host immune effector cells.
